# Comprehensive characterization of γ-aminobutyric acid (GABA) production by *Levilactobacillus brevis* CRL 2013: insights from physiology, genomics, and proteomics

**DOI:** 10.3389/fmicb.2024.1408624

**Published:** 2024-06-19

**Authors:** Pablo G. Cataldo, María Paulina Urquiza Martínez, Julio Villena, Haruki Kitazawa, Lucila Saavedra, Elvira M. Hebert

**Affiliations:** ^1^Centro de Referencia para Lactobacilos (CERELA-CONICET), San Miguel de Tucumán, Argentina; ^2^Food and Feed Immunology Group, Laboratory of Animal Food Function, Graduate School of Agricultural Science, Tohoku University, Sendai, Japan; ^3^Livestock Immunology Unit, International Education and Research Centre for Food and Agricultural Immunology (CFAI), Graduate School of Agricultural Science, Tohoku University, Sendai, Japan

**Keywords:** lactic acid bacteria, GABA synthesis, proteomics, chemically defined medium, lactobacilli

## Abstract

**Introduction:**

*Levilactobacillus brevis* CRL 2013, a plant-derived lactic acid bacterium (LAB) with immunomodulatory properties, has emerged as an efficient producer of γ-aminobutyric acid (GABA). Notably, not all LAB possess the ability to produce GABA, highlighting the importance of specific genetic and environmental conditions for GABA synthesis. This study aimed to elucidate the intriguing GABA-producing machinery of L. brevis CRL 2013 and support its potential for safe application through comprehensive genome analysis.

**Methods:**

A comprehensive genome analysis of L. brevis CRL 2013 was performed to identify the presence of antibiotic resistance genes, virulence markers, and genes associated with the glutamate decarboxylase system, which is essential for GABA biosynthesis. Then, an optimized chemically defined culture medium (CDM) was supplemented with monosodium glutamate (MSG) and yeast extract (YE) to analyze their influence on GABA production. Proteomic and transcriptional analyses were conducted to assess changes in protein and gene expression related to GABA production.

**Results:**

The absence of antibiotic resistance genes and virulence markers in the genome of L. brevis CRL 2013 supports its safety for potential probiotic applications. Genes encoding the glutamate decarboxylase system, including two gad genes (gadA and gadB) and the glutamate antiporter gene (gadC), were identified. The gadB gene is located adjacent to gadC, while gadA resides separately on the chromosome. The transcriptional regulator gadR was found upstream of gadC, with transcriptional analyses demonstrating cotranscription of gadR with gadC. Although MSG supplementation alone did not activate GABA synthesis, the addition of YE significantly enhanced GABA production in the optimized CDM containing glutamate. Proteomic analysis revealed minimal differences between MSG-supplemented and non-supplemented CDM cultures, whereas YE supplementation resulted in significant proteomic changes, including upregulation of GadB. Transcriptional analysis confirmed increased expression of gadB and gadR upon YE supplementation, supporting its role in activating GABA production.

**Conclusion:**

These findings provide valuable insights into the influence of nutrient composition on GABA production. Furthermore, they unveil the potential of L. brevis CRL 2013 as a safe, nonpathogenic strain with valuable biotechnological traits which can be further leveraged for its probiotic potential in the food industry.

## 1 Introduction

*Levilactobacillus brevis* is an obligate heterofermentative lactic acid bacteria (LAB) involved in the manufacture of a wide variety of fermented products (Cataldo et al., 2020a). Beyond their role in fermented food production, some strains of *L. brevis* also hold substantial promise as probiotics (Fang et al., 2018; Cataldo et al., 2020b; Kaur et al., 2023). Furthermore, certain metabolites produced by LAB, such as γ-aminobutyric acid (GABA), are increasingly being marketed as postbiotics constituents (Hebert et al., 2015; Torres et al., 2020; Kaur et al., 2023); offering proven health benefits to the host, including anxiety reduction and improved sleep quality (Salminen et al., 2021).

GABA, a naturally occurring and widely distributed non-proteinogenic amino acid synthesized by most living organisms, constitutes the main inhibitory neurotransmitter of the central nervous system in mammals (Walls et al., 2015). Additionally, GABA plays a crucial role in behavior, cognition, and the body’s response to stress. It also exhibits hypotensive and anti-inflammatory properties, and is known to alleviate insomnia and depression (Bajić et al., 2020; Diez-Gutiérrez et al., 2020; Cataldo et al., 2020b). Given the low natural abundance of GABA in food products, microbial fermentation emerges as a highly promising approach for producing this valuable bioactive compound. Notably, not all LAB possess the inherent ability to produce GABA, underlining the importance of both specific genetic characteristics and environmental conditions for efficient GABA synthesis (Cui et al., 2020; Cataldo et al., 2020a; Kaur et al., 2023). This production capability varies not only at the species level but also exhibits strain-dependent efficiency. In this regard, *L. brevis* strains represent the most competitive and technologically suitable bacteria used for GABA production (Cui et al., 2020; Cataldo et al., 2020a). They are generally regarded as safe and proficient in generating abundant quantities of this compound from diverse food sources.

In LAB, the biosynthesis of GABA is performed by the GAD system, which consists of the glutamate decarboxylase enzyme (encoded by *gadA* and/or *gadB*) and the glutamate: GABA antiporter encoded by *gadC* (Cui et al., 2020). The regulation of *gad* gene expression has been mainly described in *Lactococcus (Lc.) lactis* (Sanders et al., 1998; Mazzoli et al., 2010). In *Lc. lactis*, the transcriptional regulator GadR activates the expression of *gadC* and *gadB* genes in response to the presence of glutamate in the growth medium (Sanders et al., 1998). In lactobacilli, disrupting the *gadR* gene in *L. brevis* ATCC 367 impaired GABA production by reducing the expression of the *gadC* and *gadB* genes (Gong et al., 2019).

GABA production ability varies considerably among *L. brevis* strains despite possessing the same biosynthetic genetic machinery (Cui et al., 2020; Cataldo et al., 2020a; Kaur et al., 2023). Notably, *L. brevis* CRL 2013, a quinoa isolated strain, is capable of producing high levels of GABA, reaching ~265 mM GABA under optimal culture conditions in MRS supplemented with 267 mM monosodium glutamate (MSG, conversion ratio around 99%) (Cataldo et al., 2020a). These GABA levels produced by *L. brevis* CRL 2013 are regarded as the highest among those described for batch-cultured LAB strains (Gong et al., 2019; Cui et al., 2020; Cataldo et al., 2020a; Laroute et al., 2023). Previous studies revealed intriguing findings on the influence of carbon sources on GABA production by *L. brevis* CRL 2013 (Cataldo et al., 2020a). In the presence of pentoses (xylose or ribose), this strain exhibited a higher growth rate and a lower synthesis of GABA, compared to that observed in the presence of hexoses (fructose and glucose). This deficiency in GABA production was partially overcome by the addition of ethanol to the culture media (Cataldo et al., 2020a). Thus, the synthesis of GABA is not only related to the concentration of MSG present in the culture medium but is also likely influenced by the presence of specific components. *L. brevis*, like other LAB, has complex nutritional requirements, relying on carbohydrates, amino acids, salts, nucleic acid derivatives, and vitamins for optimal growth. Consequently, a chemically defined medium (CDM) is essential for accurately identifying the factors and compounds that influence GABA production. Given the limited information on the regulation of GABA synthesis in CDM by lactobacilli, this study specifically aimed to investigate the factors affecting GABA production by *L. brevis* CRL 2013 using a CDM. Through comprehensive genomic, proteomic, and transcriptional analyses, our findings enhance the understanding of GABA biosynthesis and its regulatory mechanisms in this strain.

## 2 Materials and methods

### 2.1 Microorganisms, culture media and conditions

*Levilactobacillus brevis* CRL 2013, belonging to the CERELA culture collection, was routinely grown in modified MRS broth (mMRS, Laboratorio Britania, Buenos Aires, Argentina) containing 10 g/L fructose and 10 g/L glucose (MRS-GF) as carbohydrates at 30°C for 16 h. Cells were harvested by centrifugation (8,000 × g, 15 min), washed twice with sterile 0.80% NaCl solution, and resuspended in this saline solution to the original volume. This cell suspension was used to inoculate a CDM at an initial optical density (OD) at 600 nm (OD_600_) of 0.1 (approximately 5 × 10^7^ CFU/mL). CDM was prepared according to Hebert et al. (2004) with minor modifications. The medium presented the following composition (in g/L): glucose, 10; fructose, 10; KH_2_PO_4_, 3; K_2_HPO_4_, 3; sodium acetate, 5; ammonium citrate, 1; MgSO_4_ 7H_2_O, 0.2; MnSO_4_. 4H_2_O, 0.025; L-alanine, 0.3; L-arginine, 0.3; L-asparagine, 0.6; L-aspartic acid, 0.6; L-cysteine, 0.5; L-glutamine, 0.8; L-glutamic acid, 0.8; glycine, 0.3; L-histidine, 0.3; L-isoleucine, 0.2; L-leucine, 0.2; L-lysine, 0.3; L-methionine, 0.3; L-phenylalanine, 0.3; L-proline, 0.3; L-serine, 0.3; L-threonine, 0.3; L-tryptophan, 0.2; L-tyrosine, 0.2; L-valine, 0.2; uracil, 0.01; guanine, 0.01; adenine, 0.01; nicotinic acid, 0.001; calcium pantothenate, 0.001; pyridoxal, 0.002; thiamine, 0.001; folic acid, 0.001; cyanocobalamin, 0.001; and riboflavin, 0.001. It also contained Tween 80 at 1 mL/L. All compounds were of analytical grade (Sigma- Aldrich Co.; St. Louis, MO, United States). The pH of the media was adjusted to pH 6.5 and sterilized by filtration through a sterile filter with a pore size of 0.2-μm (Gelman Sciences, Ann Arbor, MI, United States).

To determine the effect of MSG on GABA production by *L. brevis* CRL 2013, this strain was grown in CDM supplemented with 5% (w/v) MSG (CDMg). Additionally, to assess the effect of the nitrogen source on GABA production, CDMg was supplemented with yeast extract 1% (w/v) (CDMgYE). Cultures were sampled at 10 h (late exponential phase) and 36 h (stationary phase) assuming that the GAD system is activated in the stationary phase (Cataldo et al., 2020a). Culture conditions were carried out in biological triplicate.

### 2.2 GABA measurements

GABA was quantified using a modified version of the GABase method (Tsukatani et al., 2005). In the GABase assay, the following reagents were added to each well of a 96-well microtiter plate: 0.08 M Tris–HCl buffer (pH 8.9), 5 mM alpha-ketoglutarate, 3.3 mM 2-mercaptoethanol, 1.2 mM NADP^+^, and 0.03 U of GABase. The mixture was incubated at 25°C, followed by the addition of either a standard or sample solution (culture supernatants). NADPH synthesis was monitored spectrophotometrically by measurements of the OD at 340 nm every 1 min for 10 min at 25°C in a Biotek Synergy HT microplate reader (BioTek Instruments Inc., Winooski, VT, United States). GABA concentration in each sample was calculated from the calibration curve of the standard solutions (0.1, 0.25, 0.5 and 1 mM GABA).

### 2.3 Total DNA extraction, genome sequencing and assembly

Genomic DNA was extracted from CRL 2013 cells according to Brown et al. (2017). DNA concentration and purity were spectrophotometrically determined by measuring the OD_260_ and OD_280_ and determining the OD_260_/OD_280_ ratio in a Nabi- UV/Vis Nano Spectrophotometer (MicroDigital Co., Seoul, Korea). The genome was sequenced with Illumina MiSeq WGS technology. Library construction and sequencing were performed at the INDEAR sequencing facility (Rosario, Argentina). Quality control of the reads was performed using in-house programs. After this step, Illumina PCR adapter reads and low-quality reads were filtered out. The filtered reads were *de novo* assembled by Newbler v2.9 and the resulting 22 contigs were deposited in the NCBI genome database with the accession number MZMW00000000.1.

### 2.4 Bioinformatics genome characterization

Genome annotation was performed according to the standard operating procedures described by the NCBI Prokaryotic Genome Annotation Pipeline (PGAP; Annotation Software 4.10) using the best-positioned reference protein set method, GeneMarkS-2+ V

.

*L. brevis* CRL 2013 full genomic sequence was assessed for its safety application using several tools recommended in the EFSA Guidance (FEEDAP EFSA Panel, 2018); CARD (Comprehensive Antibiotic Resistance Database) for identifying antibiotic resistance (Alcock et al., 2020); and ResFinder 4.1 server^2^ for identifying the acquired antimicrobial resistance genes with a selected ID threshold of 90% and the selected minimum length of 60% and/or chromosomal mutations. Virulence factors were inferred using the RGI tool (Resistance Gene Identifier). PlasmidFinder 2.0 was used to search for mobile elements.^3^ Bacterial pathogenicity was inferred through the PathogenFinder platform (Cosentino et al., 2013). The genomic islands prediction was performed in the web-based IslandViewer4 tool using SIGI-HMM and IslandPath-DIMOB methods (Bertelli et al., 2017). The BAGEL4 web server (version 6) was utilized to predict genes encoding bacteriocins and ribosomally synthesized peptides (Van Heel et al., 2018). CRISPRFinder^4^ was used to analyze the presence of CRISPR-cas systems (Couvin et al., 2018), whereas prophage sequences were identified, annotated and graphically displayed with Phaster.^5^ Putative integrative and conjugative elements (ICE) were screened across the genome with the ICEfinder tool from the IceBerg 2.0 software (Liu et al., 2019). Integron-like elements were surveyed using the IntegronFinder tool (Galaxy Version 2.0.2 + galaxy1) available in the Galaxy platform (Néron et al., 2022).

### 2.5 Proteomic analysis

*Levilactobacillus brevis* CRL 2013 was used to inoculate basal CDM, CDMg and CDMgYE to a starting OD_600_ of 0.1. Subsequently, static incubation at 30°C was carried out, and samples were harvested at various intervals. Cell pellets were collected by centrifugation for 10 min at 8,000 x g and 4°C, then stored at −80°C.

#### 2.5.1 Sample preparation

The differential expression of intracellular proteins of the CRL 2013 strain was evaluated through a “Shot-gun bottom-up proteomics” strategy with a label-free and data dependent acquisition approach (LFQ-DDA).

Cells were harvested by centrifugation (8,000 × g, 10 min, 4°C) and washed three times with 50 mM phosphate buffer (pH 7.0). Wet pellets were mixed with glass beads (150–212 μm diameter, Sigma-Aldrich Co., St. Louis, MO, United States) and resuspended in the same buffer containing 1 mM phenylmethylsulfonyl fluoride (PMSF) and 5 mM EDTA in a 1:2:1 ratio (cells: buffer: beads). Cells were then disrupted using a Mini-BeadBeater-8 cell disruptor (Biospec Products Inc.; Bartlesville, OK, United States) at maximum speed with 10 cycles of 1 min each, with 1-min intervals on ice between cycles. To remove cell debris and glass beads, samples were centrifuged (13,000 × g, 5 min, 4°C). The protein concentration was determined according to the Bradford method (Bio-Rad Laboratories Inc., Hercules, CA, United States) using bovine serum albumin as standard. Briefly, 30 μg protein extract of each sample was reduced with 20 mM dithiothreitol for 45 min at 56°C, alkylated with 50 mM iodoacetamide for 45 min in the dark, and then digested with trypsin overnight. Extraction of peptides was performed with acetonitrile. Subsequently, samples were dried with a SpeedVac device and resuspended with 30 μL of 0.1% trifluoroacetic acid. A desalting step was carried out using Tip C18 zip columns (Merck KGaA, Darmstadt, Germany).

#### 2.5.2 LC–MS/MS data-dependent acquisition

Digested samples were analyzed by nano HPLC using an EASY-nLC 1,000 chromatograph coupled to a Q-Exactive HF mass spectrometer (Thermo Fisher Scientific, Pittsburgh, PA, United States), using an EASY-Spray Accucore (P/N ES801) Thermo Scientific reverse phase column coupled to an EASY-SPRAY Electro Spray ionizer (Thermo Fisher Scientific, Pittsburgh, PA, United States), with Spray voltage between 2.5–3.5 kV. The equipment presented a High Collision Dissociation (HCD) cell and an Orbitrap analyzer. In each cycle, the equipment performs a Full MS and then performs MS/MS on the 12 peaks with the best signal: noise ratio, with a dynamic exclusion range to reduce the number of times that the same peptide is fragmented along the cycle. Full MS Resolution: 70,000; MS/MS Resolution: 17,500; m/z range Full MS: 400–2000; AGC Target Full MS: 1e6; AGC Target MSMS: 5e5 with positive polarity.

#### 2.5.3 Protein identification

The MS spectra obtained in the previous step were analyzed with Proteome Discoverer 2.2 (Thermo Scientific), against the *L. brevis* reference pan proteome (UP000001652 UniProtKB).

In the downstream analysis, the proteins labeled as “only identified by site,” “reversed,” and “potential contaminants” were removed. The main search criteria were the following: two missed cleavages, fixed cysteine modification (carbamidomethylation), variable modifications of methionine (oxidation) and phosphorylation on threonine, serine, and tyrosine, and minimum peptide length of six amino acids. Proteome Discoverer searches were performed with a precursor mass tolerance of 10 ppm and product ion tolerance of 0.05 Da. The match between run option (0.7 min window), and the target-decoy search strategy (revert mode) options were enabled. Identifications were accepted with a false discovery rate (FDR) of 1% for peptide and proteins.

### 2.6 Real time quantitative PCR

#### 2.6.1 Total RNA extraction and cDNA synthesis

Total RNA was extracted using the Macaloid Clay method (Brown et al., 2017). The concentration of RNA was quantified spectrophotometrically in a Nabi- UV/Vis Nano Spectrophotometer (MicroDigital Co., Seoul, Korea). The DNA was removed with 5 U of TURBO™ DNase (Life Technologies Carlsbad, CA United States). The absence of genomic DNA in treated RNA samples was checked by PCR. cDNA was synthesized from 1 μg of total DNA-free RNA using random hexamer primers and Superscript III reverse transcriptase (Life Technologies Carlsbad, CA, United State) following the manufacturer instructions.

#### 2.6.2 qPCR assay

RT-qPCR was carried out on an iQ5 Real-Time PCR Detection System (Bio-Rad Laboratories Inc.) in 96-well plates using previously obtained cDNA samples as templates. Amplification products were detected using the SYBR Green fluorophore (contained in iQTM SYBR^®^ Green Supermix Kit, Bio-Rad Laboratories Inc., Hercules, CA, United States). Each reaction was performed in triplicate reaching a final volume of 20 μL containing: 10 μL 2X iQTM SYBR^®^ Green Supermix, 200 nM of each primer (Supplementary Table S1) and 30 ng of cDNA. The conditions used were: 5 min at 95°C and 40 cycles of: 1 min at 95°C, 1 min at 55°C and 30 s at 72°C, followed by a melting curve (81 cycles of 10 s starting at 5°C), which allows monitoring the dissociation kinetics of the amplified fragments. Chromosomal DNA was used as the positive control template, RNA was used as the negative control template, and for the No Template Control (NTC), no template was included. The efficiency of the resulting PCR reaction, and the threshold cycle were used for the quantification of the relative expression of the analyzed genes (Supplementary Table S1). *recA* and *rpoD* genes were used as normalization genes. For the interpretation of the relative expression results, the 2^-ΔΔCT^ method was used (Livak and Schmittgen, 2001).

### 2.7 Statistical analysis

Statistical analyses were executed with the software package Minitab 17 (Minitab Inc.) by ANOVA general linear models followed by Tukey’s *post hoc* test where *p* < 0.05 was considered significant. Unless otherwise indicated, all reported values were the means of three independent trials ± standard deviation. No significant differences were observed between individual replicates.

Proteomic data: The Perseus software V.1.6.14 was used for bioinformatics and statistical analysis (Tyanova et al., 2016). Proteins identified in all three replicates with at least 2 Peptide Spectrum Matches (PSMs) were included in the analysis. The relative differential expression analysis was performed using log_2_ transformed LFQ intensities. Proteins found to be completely absent or uniquely expressed in one condition (exclusively expressed proteins) were filtered from the pre-imputation dataset and included in the ensuing analysis as long as they met the filtering conditions for valid values (at least three valid values in the analyzed condition and completely absent in all replicates in the compared condition).

After Log2 transformation of the intensities and filtering of the data, a two-sample Student’s t-test was used to determine differentially abundant proteins with a 5% permutation-based FDR filter. For the Volcano plot construction, significant differential expression was defined as *p* ≤ 0.05 (−Log *p* ≥ 1.3) and expression difference ≥ 2 (Log_2_ expression difference ≥ 1) for upregulated proteins or Log_2_ difference ≤ −1 for downregulated proteins. Scatter plots were used to determine the correlation between replicates.

## 3 Results

### 3.1 Genome overview

#### 3.1.1 Main genomic features of *Levilactobacillus brevis* CRL 2013

The genome of *L. brevis* CRL 2013 was sequenced using Illumina MiSeq WGS massively parallel sequencing technology, achieving a coverage of 700.0x. The reads obtained were assembled *de novo* into 22 contigs. The *L. brevis* CRL 2013 genome consists of a single circular chromosome of 2,644,941 base pairs and an average guanine-cytosine (GC) content of 45.60%. Genome annotation identified a total of 38 pseudogenes and 2,654 predicted genes, including 2,581 coding DNA sequences (CDS) encoding 2,543 proteins, 6 rRNA genes, 64 tRNA genes, and 3 other non-coding RNAs. A physical genome map of *L. brevis* CRL 2013, illustrating the alignment of the contigs based on the *L. brevis* ATCC 367 reference genome, is shown in Supplementary Figure S1.

#### 3.1.2 Prediction of genetic elements related to food safety

##### 3.1.2.1 Antibiotic resistance, pathogenicity and bacteriocin production

The genome of *L. brevis* CRL 2013 was screened for known acquired resistance genes using CARD’s Resistance Gene Identifier (RGI) software with a minimum identity threshold of 75%. This initial high-stringency screening of antimicrobial resistance (AMR) databases did not identify any potential AMR genes. Further analysis with a reduced threshold stringency revealed two genes, *vanT* and *nimA* (Supplementary Table S2). The *vanT* (*locus*_tag “LBR_12095”) gene encodes an alanine racemase enzyme and *nimA* (*locus*_tag “LBR_03665”) belongs to the pyridoxamine 5′-phosphate oxidase superfamily, which is involved in vitamin B6 metabolism. Therefore, these genes are unlikely to be associated with antimicrobial resistance. ResFinder algorithm confirmed this trend depicting a non-resistant WGS-predicted phenotype against a complete list of antimicrobial substances (Supplementary Table S3).

The inquiry on the pathogenicity of the strain using the PathogenFinder tool revealed that the calculated Matched Pathogenic Families for *L. brevis* CRL 2013 was 0, and the probability of the strain to be a human pathogen was 2%. Thus, the strain was predicted as a non-human pathogen.

Regarding bacteriocin production, the analysis using BAGEL could not predict any genomic region of interest in terms of bacteriocin–related genes.

##### 3.1.2.2 Mobile genetic elements (MGE) and CRISPR/ Cas systems

Islandviewer4 (Bertelli et al., 2017) and GenoScope (Vallenet et al., 2020) were used to predict GIs in the genome of *L. brevis* CRL 2013, by sequence composition and sequence comparison, respectively. The CRL 2013 genome was aligned with the *L. brevis* ATCC 367 reference genome. Each hit from at least one algorithm (SIGI-HMM or IslandPick-DIMOB) was considered a GI. In this way, 9 islands could be identified (Supplementary Figure S2) based on codon usage (SIGI-HMM) and dinucleotide score and presence of marker genes (IslandPath-DIMOB). The identified GIs and their genes are summarized in Supplementary Table S4. Globally, the genomic islands detected by the IslandViewer4 algorithm represent 7.56% of the CRL 2013 genome with a total of 199,879 bp (Supplementary Table S4).

No integron-like elements were found within the CRL 2013 chromosome and a BLAST search against the ISfinder database resulted in 36 IS-type hits belonging mainly to the IS3, IS5, IS6, IS66, IS110, IS118, IS1202 and IS1595 families (data not shown).

Regarding the presence of elements conferring immunity to foreign genetic material, 3 CRISPR-type sequences were detected in the CRL 2013 genome in contigs 2, 3 and 12 with associated lengths of 147, 334 and 85 bp, respectively (Supplementary Table S4). Notably, no Cas-like protein coding sequences could be found. The CRISPR *locus* observed in contig 2 consists of three direct repeats (DRs) of 25 bp and two spacers of 36 bp, while the CRISPR *locus* found in contig 3 possesses six DRs of 28 bp and five spacers of 34 bp (Supplementary Figure S3). The shorter CRISPR locus on contig 12 comprises two DRs of 24 bp separated by a spacer of 37 bp (Supplementary Figure S3).

### 3.2 Genomic analysis of genes involved in GABA production

**Figure 1:**
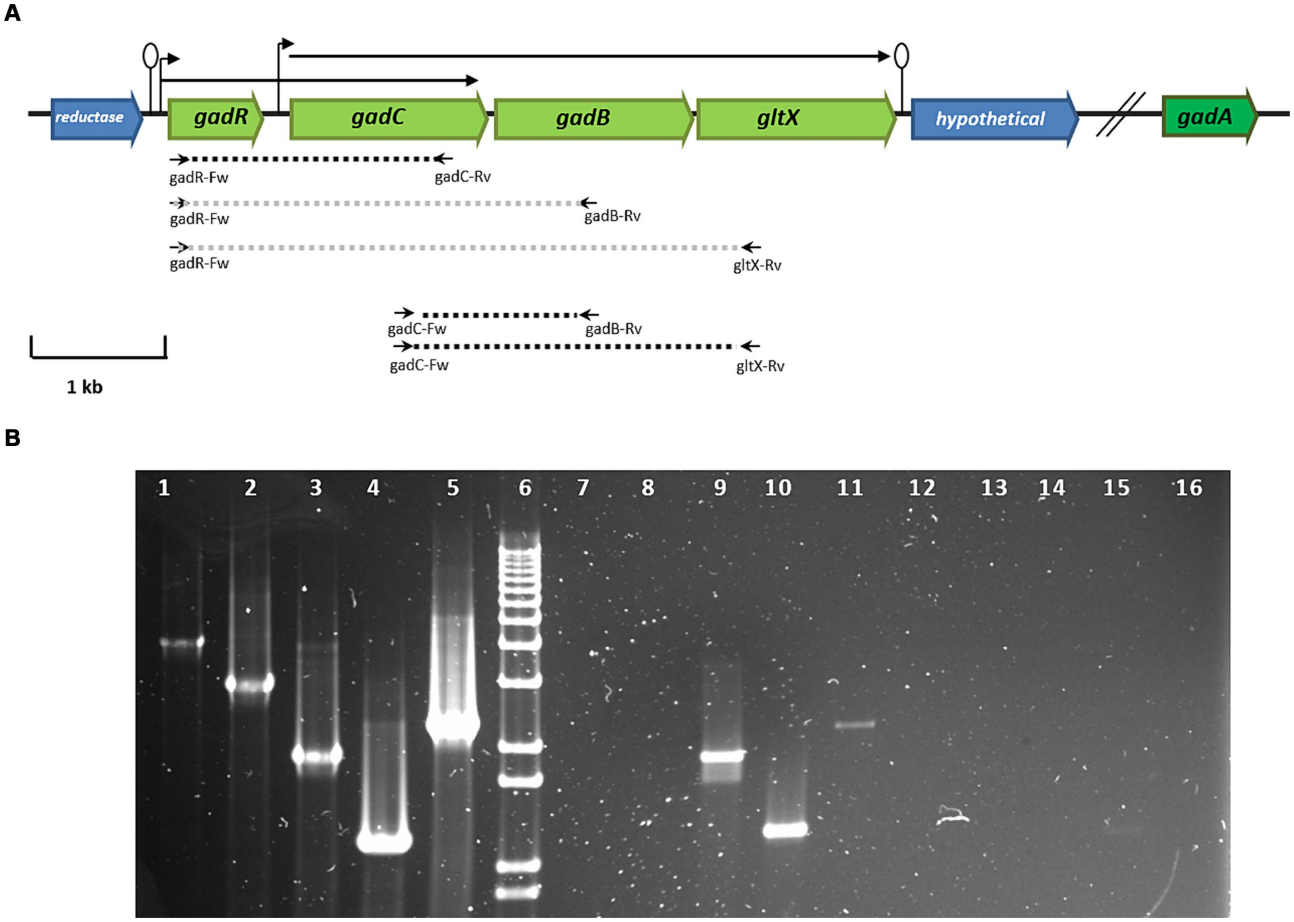
**(A)** Genetic organization of genes encoding the GAD system of *L. brevis* CRL 2013. In green, genes corresponding to the GAD system: *gadC* (glutamate: gamma-aminobutyrate antiporter), *gadR* (transcripional regulator), glutamate decarboxylases (*gadB* and *gadA*) and the glutamyl-tRNA synthetase (*gltX*) genes. Broken arrows and lollipops represent the putative promoters and terminators, respectively. The dashed lines indicate the expected size of PCR fragments using specific primers; black and gray represent amplified and non-amplified fragments, respectively, using cDNA as a template. The small horizontal arrows indicate the primers used, while the arrows drawn at the top indicate the transcriptional units found in the GAD system. **(B)** RT-PCR analysis of the transcriptional organization of GAD system genes. Lane 6, molecular size marker (1 kb plus DNA ladder, Thermo Fisher Scientific); Lanes 1, 2, 3, 4, and 5 indicate positive controls with genomic DNA as a template. Lanes 7, 8, 9, 10, and 11 show PCR amplification products using cDNA as a template. Lines 11 to 16 correspond to PCR amplification using RNA as a template. The primer pairs used were: gadR-Fw/gltX-Rv (lanes 1, 7, and 12), gadR-Fw/gadB-Rv (lanes 2, 8, and 13), gadR-Fw/gadC-Rv (lines 3, 9, and 14), gadC-Fw/gadB-Rv (lines 4, 10, and 15), and gadC-Fw/gltX-Rv (lanes 5, 11, and 16).

The genome of *L. brevis* CRL 2013 encodes two glutamate decarboxylase enzymes, GadA and GadB. It is noteworthy that the names of the two glutamate decarboxylase-encoding genes in *L. brevis* vary across different studies (Cui et al., 2020). GadA and GadB enzymes share 50.43% amino acid sequence identity (data not shown). The *gadB* gene is located adjacent to *gadC,* which encodes the glutamate:GABA antiporter. In contrast, the *gadA* gene is located separately and distantly from *gadB* on the chromosome (~ 634 kb). The transcriptional regulator gene, *gadR*, was found upstream of *gadC.* Finally, a glutamate-tRNA ligase gene, *gltX,* was located downstream of *gadB* (Figure 1). *In silico* analysis of the *L. brevis* CRL 2013 region encompassing *gad* and *gltX* genes using bacterial promoter and terminator algorithms^6^ predicted that *gadC, gadB* and *gltX* genes are cotranscribed (Figure 1). The transcriptional structure of these genes was confirmed by RT-PCR with cDNA obtained from cultures grown in both CDMg and CDMgYE, using specific primers designed to amplify intergenic regions (Supplementary Table S1 and Figure 1). Chromosomal DNA from CRL 2013 was used as the positive control template, while RNA prior to transcription into cDNA was used as the negative control template (Figure 1). PCR with the gadC-Fw/gltX-Rv primer set yielded a product of the expected size (2,285 bp), indicating that the transcript extends from *gadC* to *gltX*. Therefore, *gadC, gadB*, and *gltX* genes would be cotranscribed, belonging to a single operon (Figure 1). Furthermore, an additional transcript was observed using the gadR-Fw/gadC-Rv primer set (1,903 bp), encompassing the *gadR* and *gadC* genes, indicating the bicistronic nature of these genes (Figure 1). In contrast, no PCR product was detected when the gadR-Fw primer was combined with gadB-Rv or gltX-Rv primers (Figure 1). The absence of evidence for the cotranscription of *gadR* and *gadB* implies that they belong to separate transcriptional units. RT-PCR analysis using specific internal primers detected the *gadR, gadC, gadB*, and *gltxA* genes (data not shown), while no PCR product was detected in any of the negative control reactions (RNA without reverse transcription).

### 3.3 GABA production in a chemically defined medium

To elucidate the factors influencing GABA production by *L. brevis* CRL 2013, a CDM containing glucose and fructose as carbon sources was optimized. In this CDM medium, supplemented with 267 mM glutamate (the precursor amino acid for GABA production, CDMg), cell growth, pH and GABA production were analyzed at 30°C during 48 h. These parameters correspond to the optimal culture conditions for GABA production by *L. brevis* CRL 2013 in MRS-GF broth (Cataldo et al., 2020a). The specific growth rate of *L. brevis* CRL 2013 on glutamate-supplemented and glutamate-free CDM was similar (Table 1). Surprisingly, no GABA production or pH increase was observed in CDMg even after 48 h of incubation (Table 1), indicating that the GAD system is not active under these defined conditions. Clearly, additional nutritional factors beyond vitamins, free amino acids and MSG present in the CDM are required to stimulate GABA production by *L. brevis* CRL 2013. Previously, we demonstrated the crucial role of yeast extract (YE) supplementation in a strawberry juice for GABA production by *L. brevis* CRL 2013 (Cataldo et al., 2020b). Accordingly, CDMg was supplemented with YE (CDMgYE), resulting in a significant increase in GABA production, reaching maximum values of approximately 260 mM. This GABA increase was coupled with a rise in culture medium pH (Table 1). While final viable cell counts were comparable between CDM and CDMg (around 6.8 × 10^8^ CFU/mL), CDMgYE supported a higher final cell count (2.5 × 10^9^ CFU/mL).

**Table 1:** Effect of glutamate (CDMg) and yeast extract (CDMgYE) on cell growth, final pH, and GABA production in a chemically defined medium (CDM) by *L. brevis* CRL 2013 after 48 h of Incubation.

Culture media	Specific growth rate (h^−1^)^a^	GABA production (mM)^a^	Log CFU/mL^a^	Final pH^a^^,^^b^
CDM	0.20 ± 0.01	ND	8.83 ± 0.09	4.92 ± 0.12
CDMg	0.21 ± 0.01	ND	8.80 ± 0.10	5.00 ± 0.16
CDMgYE	0.36 ± 0.02	260.34 ± 5.11	9.41 ± 0.09	7.70 ± 0.13

a Results are presented as means ± standard deviations from at least 3 independent biological replicates. ND, not detected.

b The initial pH of all CDM was approximately 6.5.

### 3.4 Proteomic changes associated with GABA production by *Levilactobacillus brevis* CRL 2013 in CDM supplemented with glutamate

To gain deeper insights into the response of *L. brevis* to glutamate and YE supplementation, the proteomes of the CRL 2013 strain cultivated in CDM, CDMg and CDMgYE were compared.

**Figure 2:**
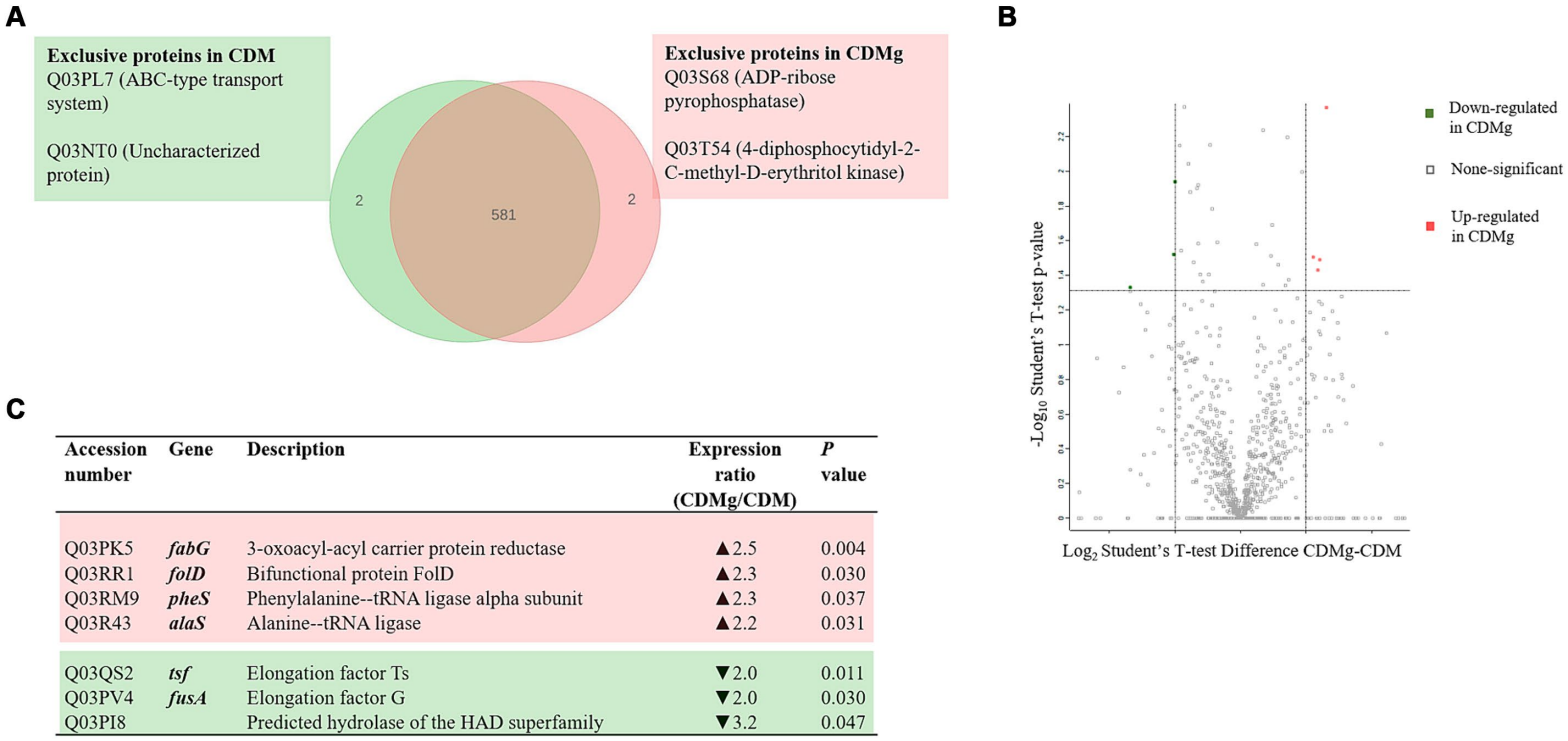
Effect of MSG on the global protein expression of *L. brevis* CRL 2013. **(A)** Venn diagram summarizing exclusive proteins in the presence (red) and absence of MSG (green). **(B,C)** Volcano Plot and table depicting significant differentially expressed proteins (DEPs) in CDMg versus CDM. Distribution of the total identified protein is shown by the volcano plot; up- and downregulated proteins in CDMg compared to CDM are shown in red and green, respectively. The dotted horizontal line represents the adjusted *p*-value threshold of 0.05, while the dotted vertical lines represent the cut-off points of difference intensities on a logarithmic scale.

A total of 583 proteins were identified in both CDM and CDMg conditions (Figure 2). Figure 2A shows proteins expressed under a unique condition, either in CDM or CDMg (exclusive proteins). The proteins expressed only in the presence of MSG were an ABC-type uncharacterized transport system protein (Q03PL7) and an uncharacterized protein (Q03NT0). In contrast, the exclusive proteins expressed in basal CDM were an ADP-ribose pyrophosphatase (Q03S68) and a 4-diphosphocytidyl-2-C-methyl-D-erythritol kinase (Q03T54) (Figure 2A). Furthermore, only four proteins were up-regulated and three were down-regulated in CDMg compared to CDM (Figure 2). This observation reflects the similarity of the proteomes under the compared conditions. Additionally, it highlights that the addition of MSG alone did not modulate any protein related to the GABA synthesis, which aligns with the undetectable GABA levels observed in the respective culture supernatants (Table 1).

### 3.5 Influence of yeast extract supplementation on GABA production

**Figure 3:**
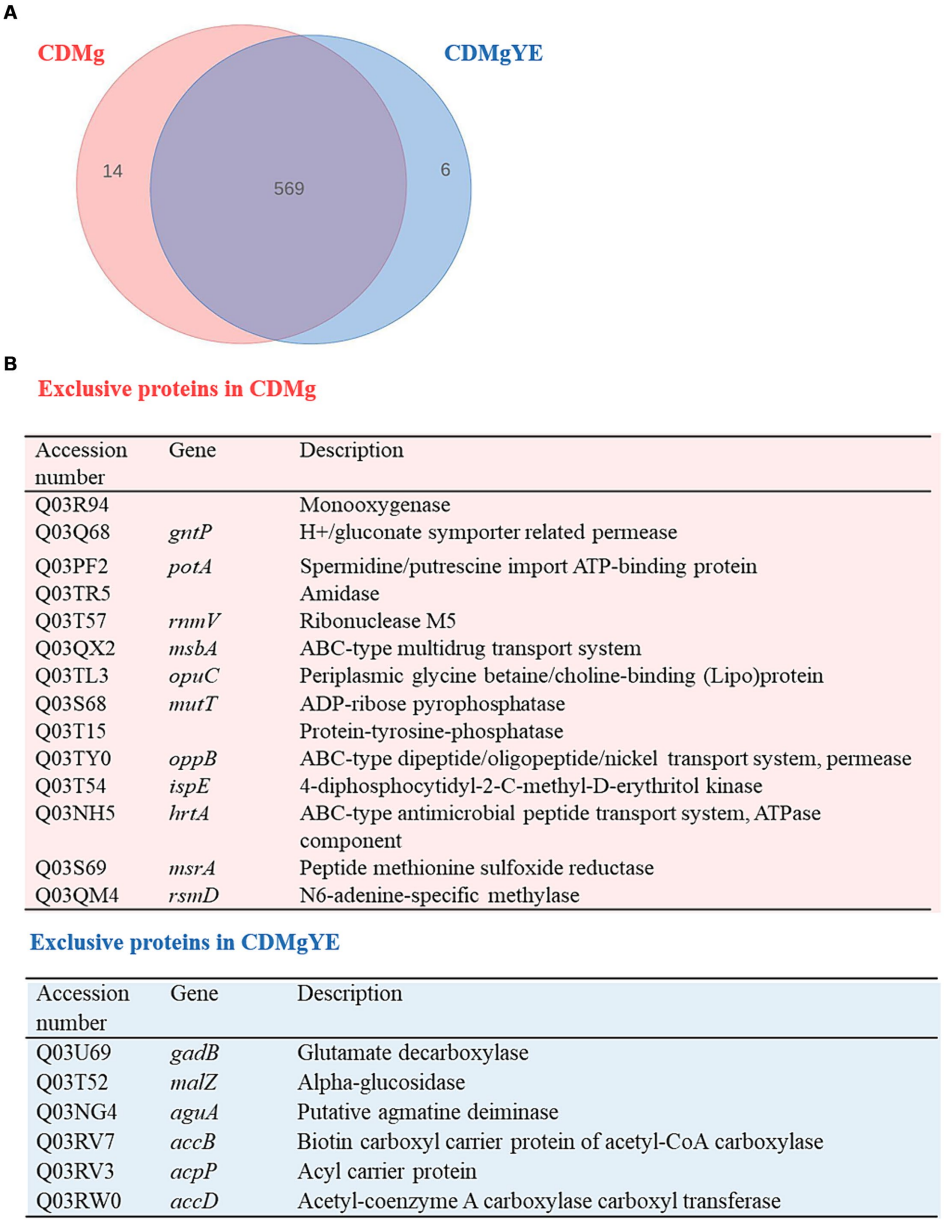
Effect of YE on the proteome of *L. brevis* CRL 2013 at exponential growth phase. **(A)** Venn diagram and **(B)** Table summarizing exclusive proteins in the presence and absence of YE.

Since GABA production was only detected in the presence of YE, the global changes in protein expression upon YE supplementation of CDMg (CDMgYE) were compared to CDMg. A total of 575 proteins were identified in the presence of YE; out of which 6 were exclusively detected in the presence of YE, while 14 proteins were only found in CDMg (Figure 3). Given the proteins detected exclusively in the presence of YE (the only condition where GABA production was observed), it is noteworthy to mention the presence of GadB (glutamate decarboxylase isoenzyme transcriptionally associated with *gadC*), involved in GABA synthesis, and agmatine deiminase (AguA). Furthermore, the total number of statistically significant DEPs was 56; with 20 being upregulated and 36 repressed in the presence of YE (Table 2). Among the proteins downregulated in the presence of YE, ten are related to oligopeptide transporter systems (Opp and Opt), which play a crucial role in the proteolytic system of LAB. In addition, the expression of proteins encoding the glutamine/ glutamate ABC transporter (GlnP, GlnQ and GlnH) were significantly downregulated in CDMgYE (Table 2).

**Table 2:** Up-regulated and down-regulated proteins in CDMgYE relative to CDMg.

Accession number	Gene	Description	Expression^a^ ratio (CDMgYE/CDMg)	*p* value
Q03U32	*adhE*	Aldehyde-alcohol dehydrogenase	▲11.4	0.002
Q03TF9	*cpnA_1*	Short-chain alcohol dehydrogenase	▲7.7	0.000
Q03TY7	*fabG*	3-oxoacyl-acyl carrier protein reductase	▲6.6	0.002
Q03PB4	*rtpR*	Adenosylcobalamin-dependent ribonucleoside-triphosphate reductase	▲5.0	0.026
Q03RY6	*pepA*	Dipeptidase	▲4.3	0.030
Q03NK2	*gpmA1*	Phosphoglycerate mutase	▲4.2	0.038
Q03RM0	*adhL*	Zn-dependent alcohol dehydrogenase	▲3.8	0.014
Q03QJ8		Amidase family enzyme	▲3.6	0.000
Q03U09	*gntK*	Gluconate kinase	▲3.2	0.047
Q03NK6	*gldA*	Glycerol dehydrogenase	▲3.2	0.006
Q03PK5	*budC*	3-oxoacyl-acyl carrier protein reductase	▲3.2	0.014
Q03U23	*ackA*	Acetate kinase	▲3.1	0.013
Q03NB5	*lacM*	Beta-galactosidase small subunit	▲3.0	0.018
Q03Q51	*deoB*	Phosphopentomutase	▲2.7	0.014
Q03NI1	*dhaT*	1,3-propanediol dehydrogenase	▲2.7	0.006
Q03NB4	*lacL*	Beta-galactosidase large subunit	▲2.6	0.018
Q03QA7	*dck*	Deoxynucleoside kinase	▲2.3	0.049
Q03QU4	*dltA*	D-alanine--D-alanyl carrier protein ligase	▲2.1	0.027
Q03R63	*ccpA*	Transcriptional regulator, LacI family	▲2.0	0.027
Q03SQ9	*groEL*	60 kDa chaperonin	▲2.0	0.028
Q03PN8	*optB*	ABC-type dipeptide/oligopeptide/nickel transport system, permease component	▼2.0	0.008
Q03SR9	*rsmI*	Ribosomal RNA small subunit methyltransferase I	▼2.1	0.046
Q03RS4	*rsmB*	tRNA and rRNA cytosine-C5-methylase	▼2.1	0.000
Q03T27	*pyrG*	CTP synthase	▼2.2	0.013
Q03TP7	*ssuE*	Predicted flavoprotein 1	▼2.2	0.048
Q03SL0	*rnr*	Ribonuclease R	▼2.3	0.047
Q03PN1	*glnq*	ABC-type polar amino acid transport system, ATPase component	▼2.3	0.037
Q03TL7	*yrdn*	4-oxalocrotonate tautomerase	▼2.5	0.000
Q03PN3	*glnp*	ABC-type amino acid transport system, permease component	▼2.6	0.006
Q03PN7	*optA*	ABC-type oligopeptide transport system, periplasmic component	▼2.6	0.005
Q03NF4	*atoB*	Acetyl-CoA acetyltransferase	▼2.6	0.026
Q03TC9	*rrf2*	Transcriptional regulator, BadM/Rrf2 family	▼2.6	0.045
Q03QY0	*glyA*	Serine hydroxymethyltransferase	▼2.7	0.002
Q03SL6	*tpiA*	Triosephosphate isomerase	▼2.8	0.012
Q03PN2	*glnH*	ABC-type amino acid transport/signal transduction system, periplasmic component/domain	▼2.9	0.011
Q03QZ7	*cnpD*	Membrane protease subunit, stomatin/prohibitin family	▼3.0	0.032
Q03PG5	*akr*	Aldo/keto reductase of diketogulonate reductase family	▼3.2	0.010
Q03QJ0	*mnmA*	tRNA-specific 2-thiouridylase	▼3.2	0.021
Q03T80	*gshR1*	Glutathione reductase	▼3.3	0.010
Q03P38	*akr*	Aldo/keto reductase of diketogulonate reductase family	▼3.6	0.010
Q03NN9	*vanX*	D-alanyl-D-alanine dipeptidase	▼3.6	0.037
Q03NW4	*gshR*	Glutathione reductase	▼3.7	0.007
Q03PP0	*optD*	ABC-type dipeptide/oligopeptide/nickel transport system, ATPase component	▼3.7	0.004
Q03PN9	*optC*	ABC-type dipeptide/oligopeptide/nickel transport system, permease component	▼3.8	0.001
Q03PP1	*optF*	ABC-type oligopeptide transport system, ATPase component	▼4.1	0.003
Q03S70	*msrB*	Peptide methionine sulfoxide reductase	▼4.4	0.029
Q03NN2	*dap2*	Dipeptidyl aminopeptidase/acylaminoacyl-peptidase	▼4.7	0.020
Q03TI3	*sad*	NAD-dependent aldehyde dehydrogenase	▼4.9	0.000
Q03TX6	*lplA*	Lipoate--protein ligase	▼5.0	0.041
Q03TX9	*oppF*	ABC-type oligopeptide transport system, ATPase component	▼6.4	0.021
Q03SF6	*mscL*	Large-conductance mechanosensitive channel	▼6.7	0.013
Q03QJ7	*yckB*	ABC-type amino acid transport system, periplasmic component	▼7.6	0.019
Q03TX8	*oppD*	ABC-type dipeptide/oligopeptide/nickel transport system, ATPase component	▼10.0	0.001
Q03PN6	*optA2*	ABC-type oligopeptide transport system, periplasmic component	▼10.4	0.000
Q03TY2	*oppA*	ABC-type oligopeptide transport system, periplasmic component	▼11.6	0.002
Q03NM1	*ywlg*	UPF0340 protein	▼21.1	0.000

a Expression levels represent the up- (▴) or down- (▾) regulation of proteins by at least two-fold in the radio CDMgYE/CDM.

### 3.6 Transcriptional analysis of genes related to GABA production

**Figure 4:**
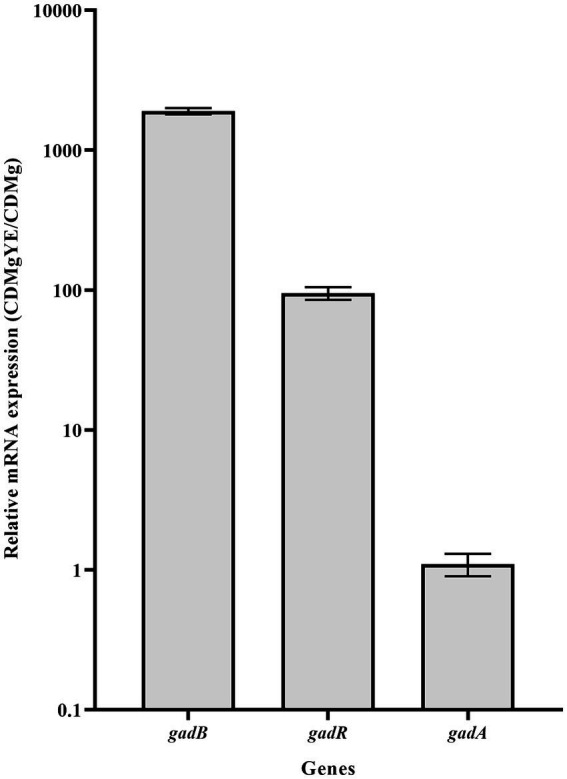
RT-qPCR analysis of the main GAD-related genes. Effect of CDMg supplementation with yeast extract (YE) on the transcription of *gadR*, *gadB* and *gadA* in *L. brevis* CRL 2013. Each bar represents the fold-change of relative mRNA expression of a gene in the respective medium relative to its expression in the basal CDMg after normalization against *recA* gene expression, using the Livak method. Means for each bar without a common letter differ significantly. Significance with Tukey’s HSD *post hoc* test following a one-way ANOVA is indicated as *p* < 0.05.

The transcriptional analysis of key genes associated with GABA production (*gadB, gadA* and *gadR*) was performed. Expression levels of *gadB* and *gadR* exhibited about 1900- and 95-fold increases in CDMgYE compared to CDMg, respectively; while *gadA* expression remained unaffected by YE supplementation (Figure 4).

## 4 Discussion

The increasing interest in LAB as probiotics highlights the need for comprehensive safety assessments due to concerns about the potential transfer of antimicrobial resistance (AMR) genes (FEEDAP EFSA Panel, 2018; Campedelli et al., 2019; Kaur et al., 2023). In our work, we used whole-genome sequencing (WGS) to evaluate the presence of potential virulence factors in the *L. brevis* CRL 2013 genome. The CARD algorithm identified only two putative AMR genes, *vanT* and *nimA*, both with low identities (32 and 48%, respectively); falling below the commonly used accepted thresholds. Further analysis revealed that the *vanT* gene is part of the alanine racemase family, essential for LAB growth (Hols et al., 1997), and not associated with the *vanG* cluster of vancomycin resistance genes (*vanU, vanR, vanS, vanXY*) (Depardieu et al., 2003). The *nimA* gene encodes an enzyme involved in vitamin B_6_ metabolism. These findings suggest a low risk of transferable AMR in *L. brevis* CRL 2013, consistent with EFSA’s guidelines exempting obligatory heterofermentative lactobacilli from vancomycin susceptibility testing (FEEDAP EFSA Panel, 2018).

WGS-based bioinformatics has become a powerful tool for understanding LAB functionality (Peng et al., 2023). Transcriptional analyses of *gad* related genes in *L. brevis* CRL 2013 identified two transcriptional units (TU); one comprising the *gadR* and *gadC* genes, and the other encompassing *gadC*, *gadB*, and *gltX* genes (Figure 1). This organization is similar to the *gadCB* operon observed in in *L. brevis* NCL912 (Li et al., 2013). Additionally, the genome of *L. brevis* CGMCC1306 showed no putative promoter or terminator signals in the intergenic region between *gadC* and *gadB*, further suggesting an operon-like structure (Lyu et al., 2018). Moreover, RNA sequencing of *L. brevis* Lbr-6108, a GABA-producing strain, revealed a cotranscription pattern for *gadR, gadC, gadB,* and *gltX* with similar read coverage across all genes (Banerjee et al., 2021). The two overlapping TUs (*gadRC* and *gadCBgltX*) observed in the CRL 2013 strain, resemble the pattern seen in Lbr-6108, suggesting a similar operon-like organization with potential regulation by different promoters within the operon (Banerjee et al., 2021). Further investigation is needed to elucidate the specific regulatory mechanisms involved.

Previous studies have shown that pH and carbon source in complex MRS broth impact GABA production by *L. brevis* CRL 2013 (Cataldo et al., 2020a). Interestingly, pentose sugars, while promoting bacterial growth, resulted in lower GABA levels compared to media containing hexoses. This deficiency was partially recovered by adding ethanol (Cataldo et al., 2020a). These findings suggest the influence of additional factors beyond the 20 amino acids present in the CDM on GABA production by *L. brevis*.

To precisely analyze the effects of different components on GABA synthesis, a CDM supporting sustained growth of *L. brevis* is necessary. Notably, this study is the first to use a CDM to investigate how other components of the medium, besides the natural 20 amino acids, influence GABA production by lactobacilli. Since there are no genes for glutamate production in *L. brevis*, an external supply of glutamate was necessary for both growth and GABA production (Lee et al., 2021). Interestingly, no GABA production was observed in CDMg; this aligns with findings for *Listeria monocytogenes,* where glutamate supplementation in a CDM did not affect its production (Karatzas et al., 2010). In contrast, *Lc. lactis* NCDO 2118 exhibited enhanced GABA production (about 10-fold) in the presence of glutamate in the CDM, converting about 5% of the glutamate to GABA after 30 h of culture (reaching a concentration of ~1.5 mM) (Mazzoli et al., 2010).

Our prior research identified *L. brevis* CRL 2013 as exhibiting the highest GABA synthesis rate and near-complete conversion of glutamate to GABA (approximately 99%) in MRS broth (a complex media containing yeast extract) (Cataldo et al., 2020a). Additionally, the importance of YE addition in a strawberry juice for GABA production by this strain was demonstrated (Cataldo et al., 2020b). Supplementing CDMg with YE resulted in GABA production levels comparable to that observed in the MRS broth (Cataldo et al., 2020a). The addition of YE to CDM significantly increased the final viable cell count compared to unsupplemented media (Table 1). However, this growth enhancement would not directly correlate with GABA production. As previously observed, pentose-containing media supported higher cell densities but resulted in lower GABA levels compared to hexose-containing media (Cataldo et al., 2020a). Furthermore, Paudyal et al. (2020) reported that CDM supplementation with nitrogen sources such as peptone or tryptone activated GABA synthesis in *Listeria monocytogenes*. Recently, Laroute et al. (2023) examined 132 strains of *Lactococcus* for GABA production in a culture medium containing glucose, YE and 34 mM glutamate; noting that GABA production constitutes a strain-dependent trait. In this medium, the NCDO 2118 strain produced about 3.8 mM GABA, with a conversion rate of ~11%; 9 times lower than that observed for *L. brevis* CRL 2013.

To deepen the understanding of the mechanisms underlying YE-enhanced GABA production, the proteomes of *L. brevis* CRL 2013 grown in different media (CDM, CDMg and CDMgYE) were compared. Glutamate supplementation alone did not induce significant changes in GABA synthesis-related proteins, corroborating the lack of GABA production observed in CDMg. Similar findings were reported for *Lc. lactis* NCDO 2118 (Mazzoli et al., 2010). While this strain exhibited a tenfold increase in GABA production when grown in a glutamate-supplemented CDM compared to the unsupplemented medium, transcriptomic and 2-DE analyses failed to identify any upregulated glutamate decarboxylase enzymes among the overexpressed cytosolic or membrane-associated proteins (Mazzoli et al., 2010).

Proteomic analysis of CDMgYE revealed the upregulation of GadB, a protein directly involved in GABA synthesis, and AguA, an enzyme involved in agmatine conversion and ammonium release. These two proteins are part of metabolic pathways associated with acid resistance (Banerjee et al., 2021). The expression of GadA did not significantly change under any condition. Both proteomic and transcriptional results indicated that YE triggers the upregulation of GadB, highlighting the role of this glutamate decarboxylase in enhancing GABA production. Consistent with these findings, studies performed in *L. brevis* CGMCC1306, involving deletions in *gad* genes, revealed that GAD activity and GABA production were only affected after the deletion of *gadB* (Lyu et al., 2018). Furthermore, Gong et al. (2019) demonstrated that *gadCB* expression is transcriptionally regulated by its activator *gadR*. This expression pattern was also observed in CRL 2013 cells grown in CDMgYE, emphasizing the role of GadR as a key positive transcriptional regulator governing *gadCB* expression and GABA production in *L. brevis* CRL 2013.

Additionally, *L. brevis* CRL 2013 grown with YE supplementation exhibited a decrease in the expression of proteins associated with oligopeptide transport systems (Opp and Opt). The proteolytic system of LAB consists of three major components; a cell envelope-associated proteinase (CEP), oligopeptide transporter systems, and several intracellular peptidases. *L. brevis* CRL 2013 lacks CEP and possesses two oligopeptide transport systems, the *oppDFBCA* and *optABCDF* operons (data not shown). LAB closely regulate their proteolytic systems based on nitrogen availability within the cell, (Savijoki et al., 2006; Brown et al., 2017; Elean et al., 2020). The absence of repression of the oligopeptide systems in CDMg, which contains the 20 amino acids, compared to CDMg, YE might be due to the low efficiency of amino acid uptake, limiting the availability of branched-chain amino acids inside the cell (Guedon et al., 2001a,b). This limitation prevents the complete repression of gene expression by transcriptional repressors like CodY in *Lactococcus* (Guedon et al., 2001a,b). The addition of peptides, which are taken up more efficiently, overcomes this low uptake efficiency (Guedon et al., 2001b; Hebert et al., 2008). Proteomic and transcriptional approaches in several LAB have extensively demonstrated the downregulation of several components of the proteolytic system, including the Opp and Opt systems, in the presence of a peptide supply in the growth medium (Gitton et al., 2005; Savijoki et al., 2006; Brown et al., 2017). In *Lactobacillus delbrueckii* subsp. *lactis* CRL 581, the addition of Casitone (a rich source of peptides) to a CDM resulted in a 5- to 29-fold repression of some components of the Opp and Opt transport systems (Brown et al., 2017). Similarly, a proteomic approach in *Lc. lactis* NCDO 763 revealed that the levels of OppA, OppD, and OptF were significantly increased upon growth in a medium without peptide supply (Gitton et al., 2005).

In conclusion, this research presents significant novelties in the study of *L. brevis* CRL 2013 by using a CDM to investigate the effects of glutamate and yeast extract on GABA production. The study revealed a unique transcriptional organization of the glutamate decarboxylase system genes and identified key metabolic modulations induced by YE. These findings offer valuable insights into the safe application of *L. brevis* CRL 2013 for efficient GABA production in the food industry, addressing safety concerns related to antimicrobial resistance while advancing our understanding of LAB functionality.

## Data availability statement

The datasets presented in this study can be found in online repositories. The names of the repository/repositories and accession number(s) can be found at: https://www.ncbi.nlm.nih.gov/genbank/, MZMW00000000.1.

## Author contributions

PC: Conceptualization, Formal analysis, Investigation, Methodology, Writing – review & editing, Data curation, Visualization, Writing – original draft. MU: Methodology, Writing – review & editing. JV: Methodology, Writing – review & editing, Formal analysis. HK: Conceptualization, Funding acquisition, Project administration, Resources, Supervision, Writing – review & editing. LS: Conceptualization, Project administration, Resources, Supervision, Writing – review & editing. EH: Formal analysis, Methodology, Writing – review & editing, Conceptualization, Funding acquisition, Investigation, Project administration, Supervision, Validation.

## Footnotes

^2^https://cge.cbs.dtu.dk/services/ResFinder

^3^https://cge.cbs.dtu.dk/services/PlasmidFinder-2.0/

^4^https://crisprcas.i2bc.paris-saclay.fr/CrisprCasFinder/Index

^5^https://phaster.ca/

^6^http://www.softberry.com
