# Supplementary material for: Comprehensive characterization of γ-aminobutyric acid (GABA) production by Levilactobacillus brevis CRL 2013: insights from physiology, genomics, and proteomics
Source: Front Microbiol. 2024 Jun 19;15:1408624. doi: 10.3389/fmicb.2024.1408624 (PMC11219586; doi:10.3389/fmicb.2024.1408624)
Supplement: Supplementary file 1 [file Table_1.DOCX]

**Supplementary Table S1.** Primers used in this study

| **Gene or target (locus)** | **Predicted function** | **Primer sequence (5´to 3’)** | **Amplicon length (bp)** |  |
| --- | --- | --- | --- | --- |
| **RT-qPCR analyses** | | | | |
| *rpoD*  (LBR_08040) | RNA polymerase sigma factor RpoD | *rpoD Fw* TTGGCCTAGATGATGGTCGG  *rpoD Rv* GTTTACTGCGTGATGGGTGC | 129 | |
| *recA*  (LBR_10325) | DNA recombination/repair protein RecA | *recA Fw* GCCTTGATTTCCAGTGGTGC  *recA Rv* GTGACATCAATCGGGCTTGC | 127 | |
| *gadB*  (LBR_00015) | Glutamate decarboxylase | *gadB Fw*: GTTGATGCTGCCTTTGGTGG  *gadB Rv*: GCCAAACAATCCACCCCAAC | 145 | |
| *gadC*  (LBR_00020) | Glutamate-GABA antiporter | *gadC Fw:* CGGCCTTTGGATTTGTCGTG  *gadC Rv:* GCAAAACGAGCATGACCAC | 112 | |
| *gadR*  (LBR_00025) | Transcriptional regulator | *gadR Fw:*GTCGATTCCCATGCTTATTCG  *gadR Rv:* GCGGAAATGTAACTGCGGAC | 115 | |
| *gadA*  (LBR_03025*)* | Glutamate decarboxylase | *gadA Fw:* GCTTCCGGCGGATTTTACAC  *gada Rv:* CAGATTACCCAGCCGACTCC | 134 | |
| **Transcriptional analyses** | | | | |
| *gadRCB-gltx* |  | *gadR Fw:*GTCGATTCCCATGCTTATTCG  *gltX-Rv* TGGTTCGCCGGCAAATGGAGG | 4,076 | |
| *gadRCB* |  | *gadR Fw:*GTCGATTCCCATGCTTATTCG  *gadB Rv*: GCCAAACAATCCACCCCAAC | 2,971 | |
| *gadRC* |  | *gadR Fw:*GTCGATTCCCATGCTTATTCG  *gadC Rv:* GCAAAACGAGCATGACCAC | 1,903 | |
| *gadCB* |  | *gadC-Fw* CGGCCTTTGGATTTGTCGTG  *gadB Rv*: GCCAAACAATCCACCCCAAC | 1,180 | |
| *gadCB-gltX* |  | *gadC-Fw* CGGCCTTTGGATTTGTCGTG  *gltX-Rv* TGGTTCGCCGGCAAATGGAGG | 2,285 | |
