# Supplementary material for: Comprehensive characterization of γ-aminobutyric acid (GABA) production by Levilactobacillus brevis CRL 2013: insights from physiology, genomics, and proteomics
Source: Front Microbiol. 2024 Jun 19;15:1408624. doi: 10.3389/fmicb.2024.1408624 (PMC11219586; doi:10.3389/fmicb.2024.1408624)
Supplement: Supplementary file 2 [file Table_2.DOCX]

**Supplementary Table S2** Summary of CARD-RGI results

| **RGI Criteria** | **ARO Term** | **Start** | **End** | **Strand** | **Detection criteria** | **AMR gene family** | **Drug**  **class** | **Resistance mechanism** | **% Identity of matching region** | **% Length of reference sequence** |
| --- | --- | --- | --- | --- | --- | --- | --- | --- | --- | --- |
|  |  |  |  |  |  |  |  |  |  |  |
|  |  |  |  |  |  |  |  |  |  |  |
|  |  |  |  |  |  |  |  |  |  |  |
| Strict | *nimA* | 778654 | 779139 | + | protein homolog model | nitroimidazole reductase | nitroimidazole antibiotic | antibiotic inactivation | 47.56 | 91.48 |
| Strict | *vanT* gene in *vanG* cluster | 98370 | 99491 | + | protein homolog model | glycopeptide resistance gene cluster, vanT | glycopeptide antibiotic | antibiotic target alteration | 31.82 | 52.39 |
