# Supplementary material for: Comprehensive characterization of γ-aminobutyric acid (GABA) production by Levilactobacillus brevis CRL 2013: insights from physiology, genomics, and proteomics
Source: Front Microbiol. 2024 Jun 19;15:1408624. doi: 10.3389/fmicb.2024.1408624 (PMC11219586; doi:10.3389/fmicb.2024.1408624)
Supplement: Supplementary file 5 [file Image_1.PDF]

Supplementary figures

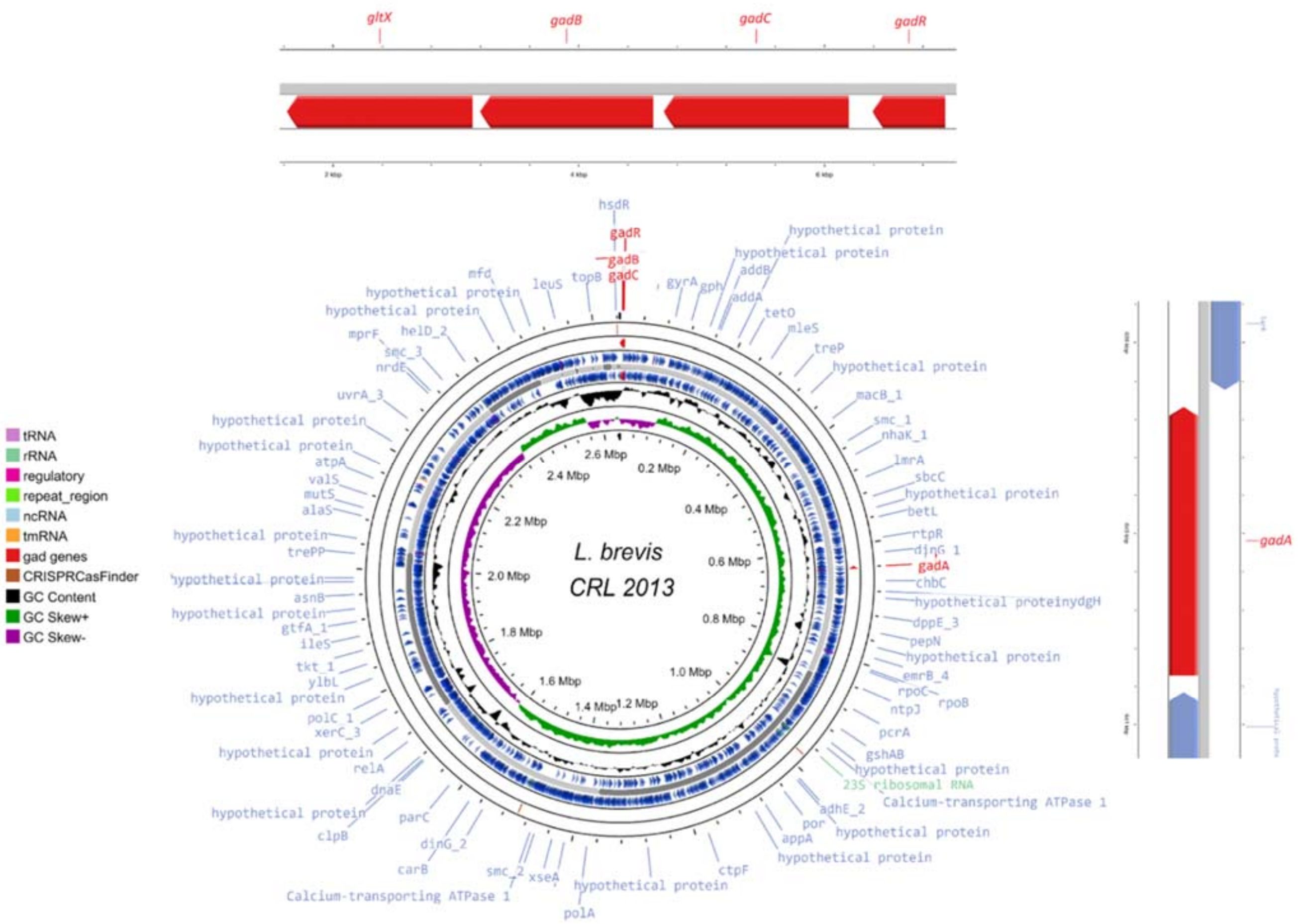

Supplementary Figure S1. Circular map view of the *L. brevis* CRL 2013 genome.

The contents are arranged in feature rings (starting with outermost ring): outermost first ring represents the mapped CRISPR elements. The second ring represents the identified *gad* genes. Third to fifth tracks represent the 22 aligned contigs flanked by the CRL 2013 coding sequences (CDS) on the leading and lagging strands; the two inner-most tracks display the G+ C content (black) and G/C skew information (green and violet).

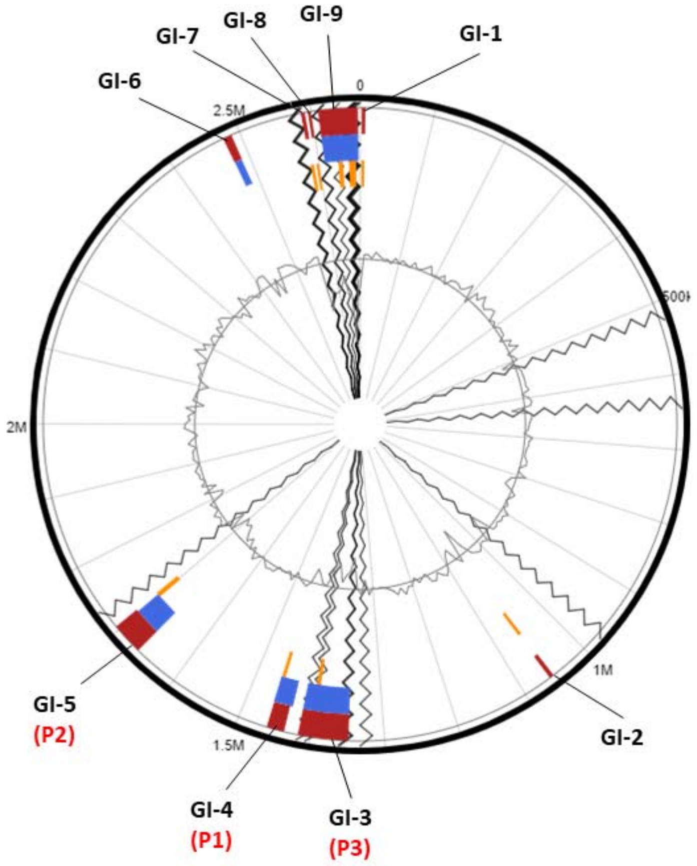

Supplementary Figure S2. Predicted GIs and prophage regions in the genome of *L. brevis* CRL 2013 after alignment against reference genome *Levilactobacillus brevis* ATCC 367. Gaps are indicated as grey bars in the circular plot, and unaligned contigs are displayed at the end of the genome plot (indicated in grey on the circular alignment plot). The colored shapes orange, blue and red represents the predicted genomic islands as identified by SIGI-HMM (orange) and IslandPath-DIMOB (blue), and red showing the integrated genomic island search results.

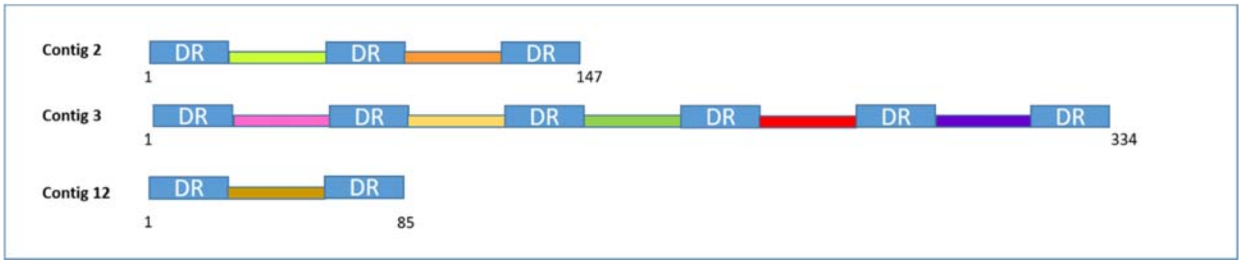

Supplementary Figure S3. CRISPR structures were identified in the genome of *L. brevis* CRL 2013 using the *CRISPRFinder* platform. Repeats are highlighted in blue boxes, while spacers are shown in distinct colors. The numbers indicate the length (in base pairs) of the CRISPR structures found on each contig.
